# Supplementary material for: Blockade of HVEM for Prostate Cancer Immunotherapy in Humanized Mice
Source: Cancers (Basel). 2021 Jun 16;13(12):3009. doi: 10.3390/cancers13123009 (PMC8235544; doi:10.3390/cancers13123009)
Supplement: Supplementary file 1 [file cancers-13-03009-s001.zip › cancers-1238626-supplementary.pdf]

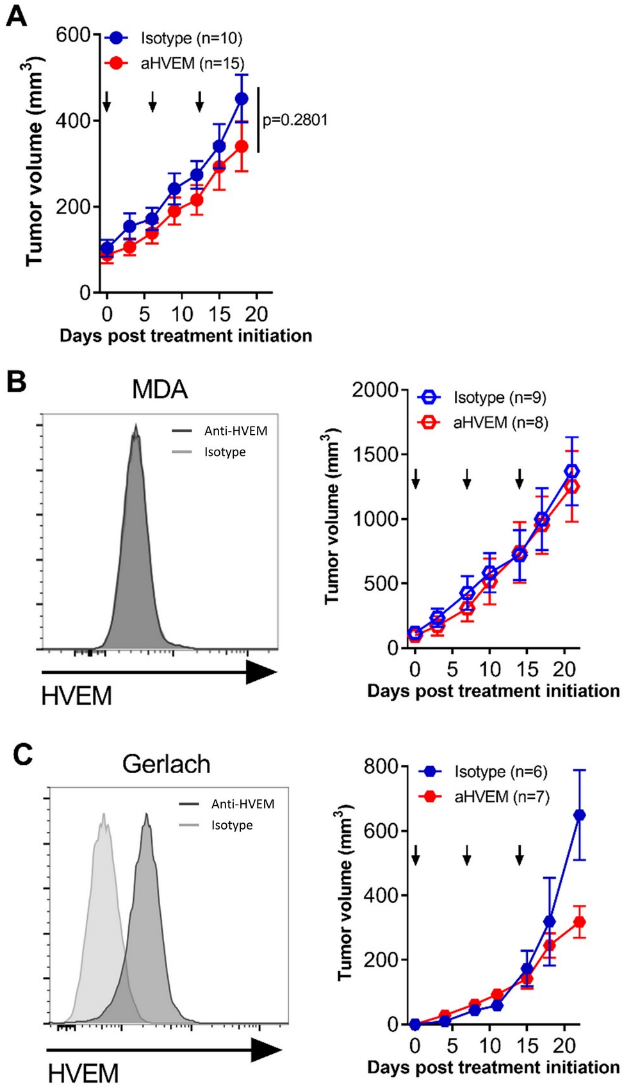

Figure S1: Anti-HVEM therapy in humanized mice. (A) Mean tumor volume (± SEM) of the PC3 prostate cancer cell line in non-humanized NSG mice at the indicated time after treatment initiation. Arrows indicates days of the injection of the anti-HVEM mAb. Number of mice are indicated in brackets. The p value is the result of a linear regression analysis testing the null hypothesis that the two slopes are identical. Results are compiled from 3 independent experiments. **(B)** Expression of HVEM on the MDA-MB-231 breast cancer cell line (MDA) by flow cytometry. Pale grey histogram is from the FMO control. Mean tumor volume (± SEM) of MDA-MB-231 cells in humanized mice treated at the indicated times with the anti-HVEM mAb (arrows). Number of mice included in the study are indicated in brackets. Results are compiled from 2 independent experiments. **(C)** Expression of HVEM on the Gerlach melanoma cell line by flow cytometry. Pale grey histogram is from the FMO control. Mean tumor volume (± SEM) of Gerlach cells in humanized mice treated at the indicated times with the anti-HVEM mAb (arrows). Number of mice included in the study are indicated in brackets. Results are compiled from 2 independent experiments.

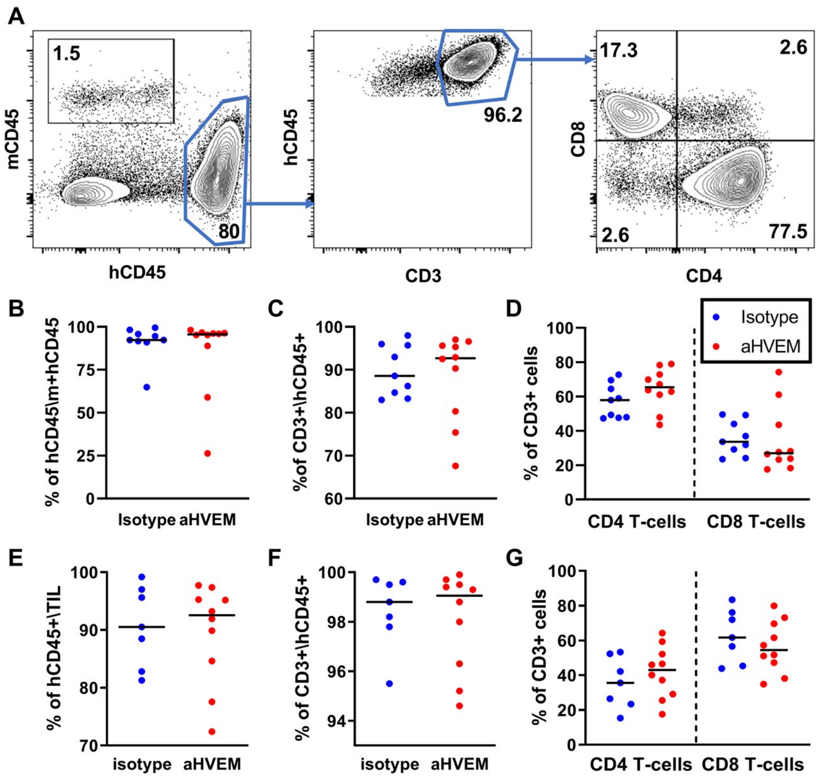

Figure S2: Human cell reconstitution in spleen and PC3 tumor of humanized mice. (A) Representative dot plot showing the gating strategy to determine the frequencies of human cells in the spleens of humanized mice. **(B)** Frequencies of human $CD45^+$ cells among total $CD45^+$ cells (murine + human (m+h)) from spleens of humanized mice **(C)** Frequencies of $CD3^+$ cells among human $CD45^+$ cells. **(D)** Frequencies of $CD4^+$ or $CD8^+$ cells in $CD3^+CD45^+$ cells. Same results for **(E)** $CD45^+$, **(F)** $CD3^+$, **(G)** CD4 and CD8 T-cells from PC3 tumors are shown. Each dot is a mouse. Results were obtained at D24 after transfer of human PBMCs and are cumulative of 2 independent experiments.

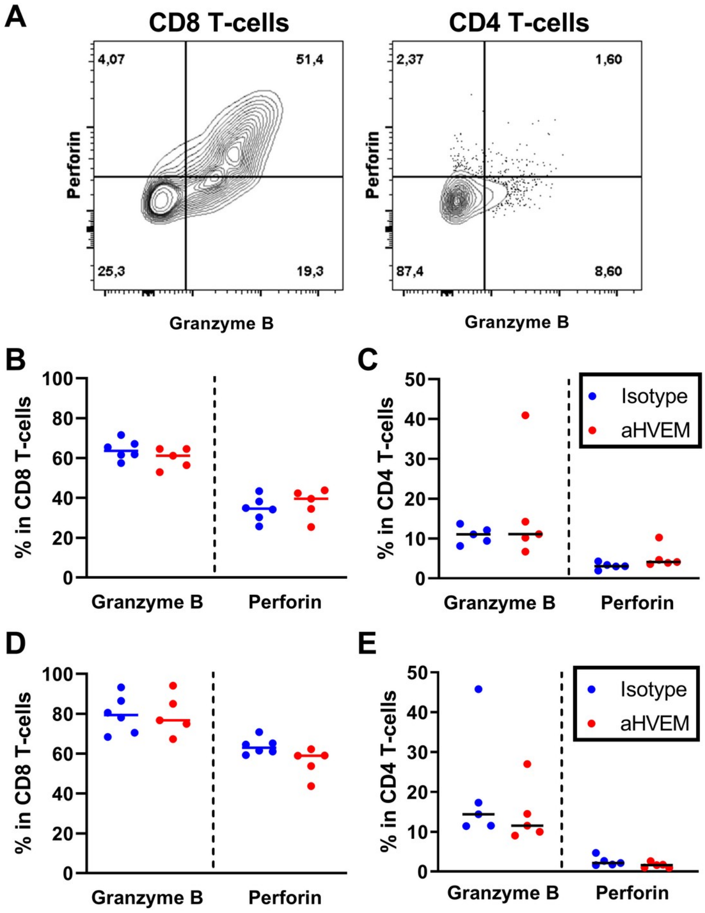

Figure S3 : Granzyme B and Perforin expression and frequencies in T-cells of humanized mice. (A) Representative contour plot of granzyme B and perforin expression in CD8^{+} and CD4^{+} T-cells from PC3 tumor. **(B-E)** Cumulative frequencies of granzyme B and perforin without ex vivo stimulation in CD8^{+} and CD4^{+} T-cells in spleens **(B, C)** or PC3 tumors **(D, E)** of humanized mice at day 21 after treatment initiation. Each dot is a mouse. Data are from one experiment.

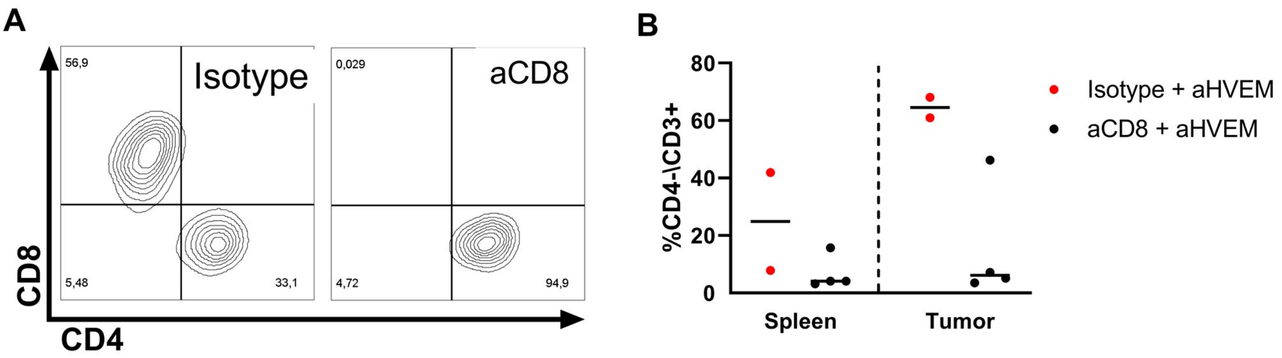

Figure S4: CD8 depletion efficiency in humanized mice. (A) Representative contour plot of CD4 and CD8 staining in CD45/CD3$^+$ T-cells from the spleen of humanized mice treated with isotype or anti CD8 depleting reagent (aCD8). **(B)** Frequencies of CD4$^-$ in CD3$^+$ T-cells in spleens and PC3 tumors of humanized mice at day 24 post CD8 depletion. All mice were treated with anti-HVEM (aHVEM). Each dot is a mouse. Data are from one representative experiment out of two.

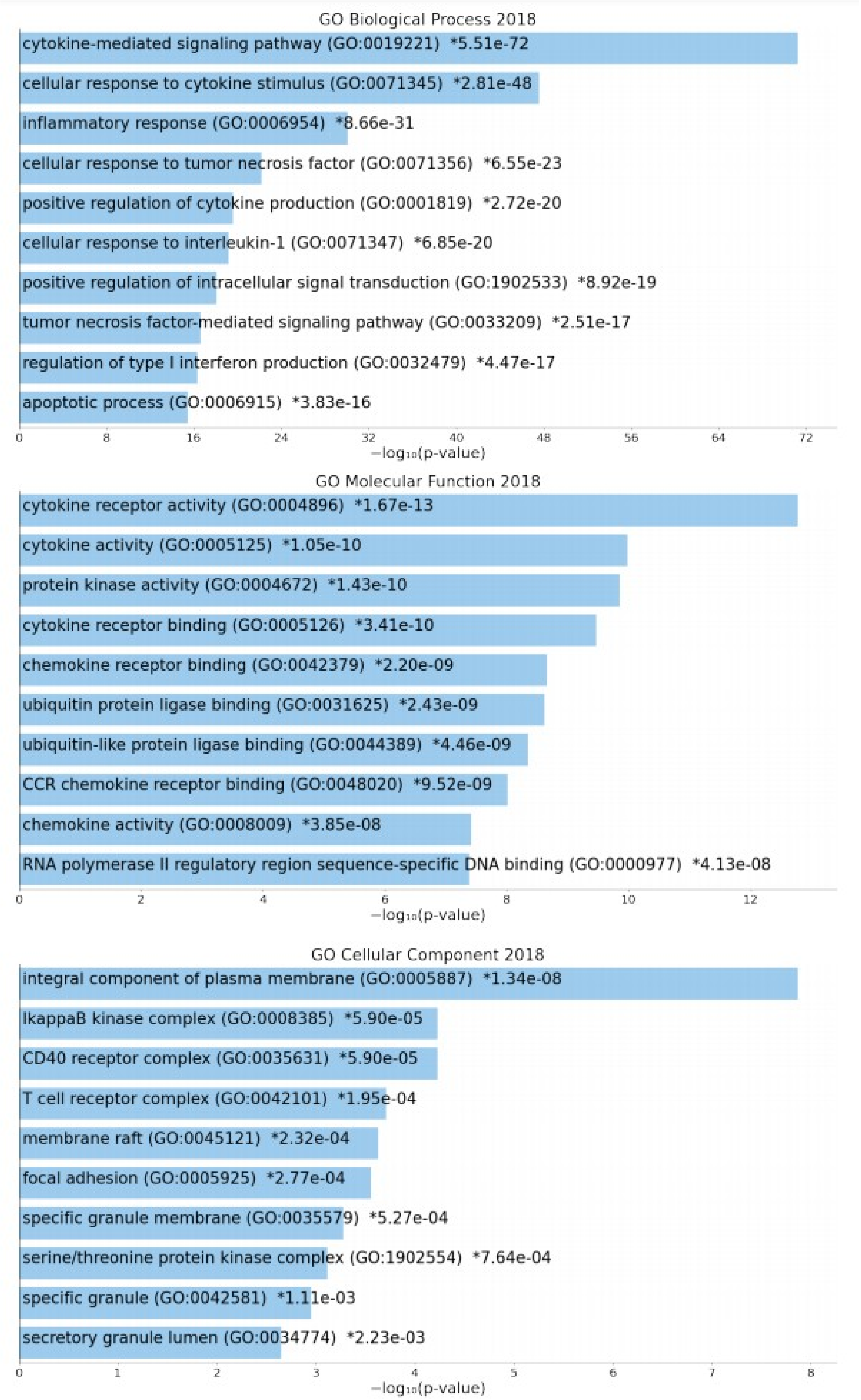

Figure S5: Enrichment analysis in TILs of anti-HVEM-treated mice. A list of DEG up regulated by the antiHVEM treatment in CD45+ TILs was entered in Enrichr and analyzed with Appyter. The top 10 ontologies in each of the indicated categories (Biological process, Molecular function and Cellular component) are represented according to their p value from an exact Fisher test embedded in the application. A star denotes an adjusted p value inferior to 0.05.

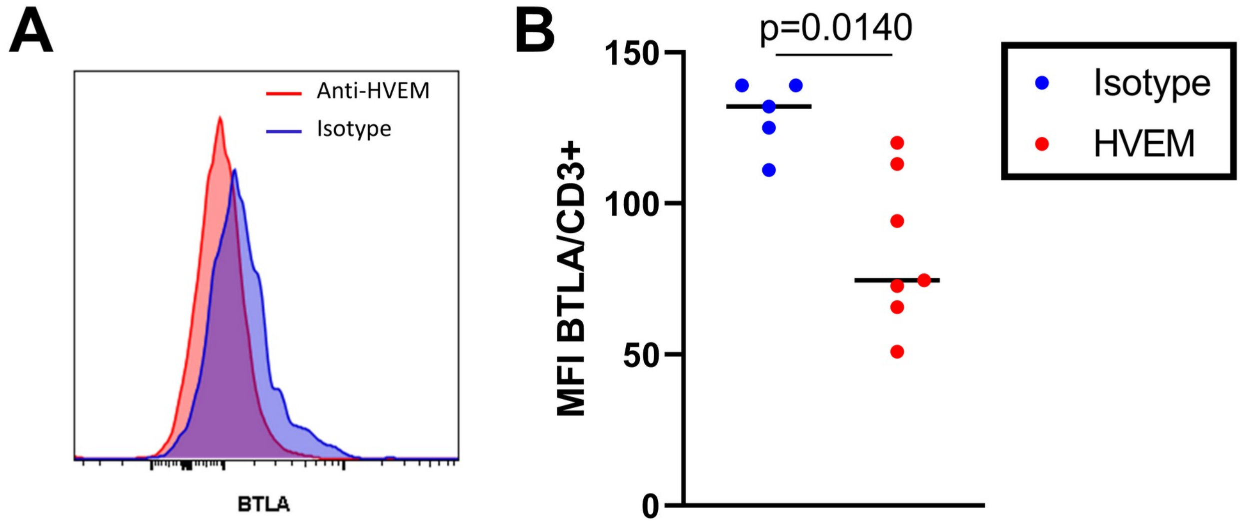

Figure S6 : BTLA expression in CD3+ TILs from PC3 tumors of humanized mice. (A) Representative expression of BTLA expression in anti-HVEM- or isotype-treated mice. **(B)** Median fluorescence intensity (MFI) of BTLA at day 21 post-humanization. Each dot is a mouse. Data are from one representative experiment out of two. The p values are from a Mann-Whitney t test.
